# The relative contributions of climate, soil, diversity and interactions to leaf trait variation and spectrum of invasive *Solidago canadensis*

**DOI:** 10.1186/s12898-019-0240-1

**Published:** 2019-06-15

**Authors:** Li-Jia Dong, Wei-Ming He

**Affiliations:** 1 State Key Laboratory of Vegetation and Environmental Change, Institute of Botany, Chinese Academy of Sciences, Beijing, 100093 China; 2 College of Life Science, Shaoxing University, Zhejiang, 312000 China; 3 College of Resources and Environment, University of Chinese Academy of Sciences, Beijing, 100049 China

**Keywords:** Climate, Invader–community interactions, Invasive plants, Multimodel inference, Native plant diversity, Soil properties

## Abstract

**Background:**

Invasive plants commonly occupy diverse habitats and thus must adapt to changing environmental pressures through altering their traits and economics spectra, and addressing these patterns and their drivers has an importantly ecological and/or evolutionary significance. However, few studies have considered the role of multiple biotic and abiotic factors in shaping trait variation and spectra. In this study, we determined seven leaf traits of 66 *Solidago canadensis* populations, and quantified the relative contributions of climate, soil properties, native plant diversity, and *S. canadensis*–community interactions (in total 16 factors) to leaf trait variation and spectrum with multimodel inference.

**Results:**

Overall, the seven leaf traits had high phenotypic variation, and this variation was highest for leaf dry matter content and lowest for leaf carbon concentration. The per capita contribution of climate to the mean leaf trait variation was highest (7.5%), followed by soil properties (6.2%), *S. canadensis*–community interactions (6.1%), and native plant diversity (5.4%); the dominant factors underlying trait variation varied with leaf traits. Leaf production potential was negatively associated with leaf stress-tolerance potential, and the relative contributions to this trade-off followed in order: native plant diversity (7.7%), climate (6.9%), *S. canadensis*–community interactions (6.2%), and soil properties (5.6%). Climate, diversity, soil, and interactions had positive, neutral or negative effects.

**Conclusions:**

Climate, soil, diversity, and interactions contribute differentially to the leaf trait variation and economics spectrum of *S. canadensis*, and their relative importance and directions depend on plant functional traits.

**Electronic supplementary material:**

The online version of this article (10.1186/s12898-019-0240-1) contains supplementary material, which is available to authorized users.

## Background

Plants, in particular those with widespread distribution, occupy diverse habitats and therefore experience contrasting selective pressures (e.g., variable climatic conditions, soil resources, organisms, and stresses) [1–5]. Consequently, their performance is tightly linked to a suite of biotic and abiotic factors [1–3, 6, 7]. In nature, plants can respond to diverse habitats and selective pressures in many ways. Two of these, changes in plant traits alone (i.e., trait variation) and relationships among plant traits (i.e., trait spectra) have been acknowledged as effective ways through which plants can maximize their fitness. Thus, they have received increasing attention over the past decades [8–15].

Studying plant traits along ecological gradients commonly focuses on two points: trait variation and its drivers; trait spectra and their drivers. Addressing these patterns and their drivers has an importantly ecological and/or evolutionary significance. For example, trait variation can reflect selective pressures and evolutionary trajectories [2, 16] and trait spectra can be incorporated into the ecological strategies of plants to adapt to changing environments [9, 10]. However, several aspects remain poorly understood. First, previous studies have primarily addressed the role of abiotic factors (e.g., climatic, edaphic, and topographic gradients) in shaping plant traits [9, 17–19]. Accordingly, few previous studies have addressed the role of biotic factors, in particular the relative importance of biotic and abiotic factors in shaping trait variation and spectra. Second, most of the related studies have usually involved different species. Such cross-species studies encompass phylogenetic effects and thus identifying ecological effects is difficult [19], and examining trait variation and spectra among populations within the same species might help to better understand them mechanistically [20].

Invasive plants provide an ideal stage for understanding trait variation and spectra along ecological gradients. First, plant invaders can expand their range rapidly and thus occupy diverse habitats and face different environmental pressures [4, 5, 21]. Second, plant invaders commonly have a growth advantage [22]. This advantage is closely linked to high variation of phenotypic traits so as to obtain essential resources [23]. Third, plant invaders are likely to have relatively low genetic variation due to asexual/clonal reproduction [19], thereby highlighting ecological effects. Additionally, there are strong interactions between plant invaders and recipient plant communities [24, 25].

The aim of this study was to examine trait variation and trait spectrum among invasive plant populations along multiple gradients and to quantify the relative contributions of multiple biotic and abiotic factors. More specifically, we focused on leaf traits for the following reasons: leaf traits (e.g., leaf thickness, size, chlorophyll, and stoichiometry) can control key ecological functions (e.g., carbon [C] economy, tolerance to stresses, and nutrient cycling) and reflect the strategies of plants to cope with contrasting selective pressures [26]. Leaves face two basic challenges (i.e., C fixation and stress tolerance) during their lifespan, and leaf production potential and stress-tolerance potential are convergent in common gardens (i.e., a positive relationship between production potential and tolerance potential in the same conditions) [26]. Thus, we hypothesized that this convergent relationship between production potential and tolerance potential might occur in successful plant invaders. However, it should be noted that negative relationships between leaf production and leaf tolerance can also occur along environmental gradients [26].

To achieve the above purpose, we investigated 66 populations of an invader, *Solidago canadensis,* and determined seven leaf traits and 16 biotic and abiotic variables per population. These variables play a key role in determining the responses of plants to changing environments and could be categorized into four categories: climate (two variables), native plant diversity (two variables), soil properties (six variables), and *S. canadensis*–community interactions (six variables). Specifically, we attempted to address the following two questions. (i) How do different variables contribute to the variation in seven leaf traits? (ii) How do different variables contribute to a trait spectrum (i.e., the relationship between leaf production potential and leaf stress-tolerance potential)?

## Results

**Fig. 1:**
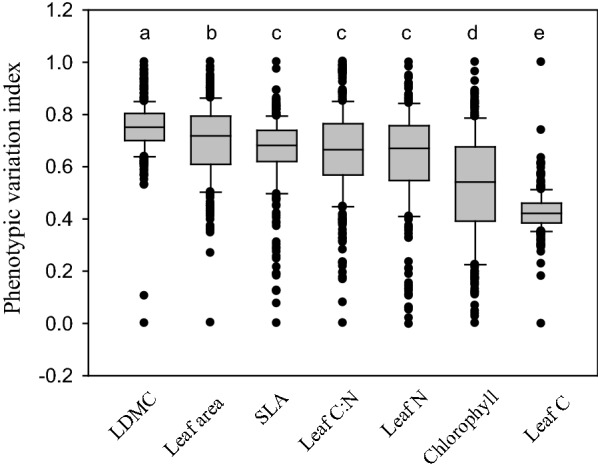
The boxplot of phenotypic variation index of seven leaf traits. LDMC, leaf dry matter content; SLA, specific leaf area. The different letters indicate significant differences at the *P* = 0.05 level

The phenotypic variation index (PVI) of leaf dry matter content (LDMC) was highest among the seven leaf traits measured in this study (Fig. 1: PVI = 0.749 ± 0.006 [1 SE]). The PVI of leaf area (PVI = 0.696 ± 0.008) was lower than that of LMDC but higher than that of the other five leaf traits (Fig. 1). Specific leaf area (SLA: PVI = 0.656 ± 0.008), leaf C:N ratio (PVI = 0.655 ± 0.009), and leaf N concentration (PVI = 0.634 ± 0.011) shared the same PVI values, and they had greater variation than leaf chlorophyll (PVI = 0.522 ± 0.0121) and leaf C concentration (PVI = 0.427 ± 0.005); the PVI of leaf chlorophyll was higher than that of leaf C concentration (Fig. 1).

**Fig. 2:**
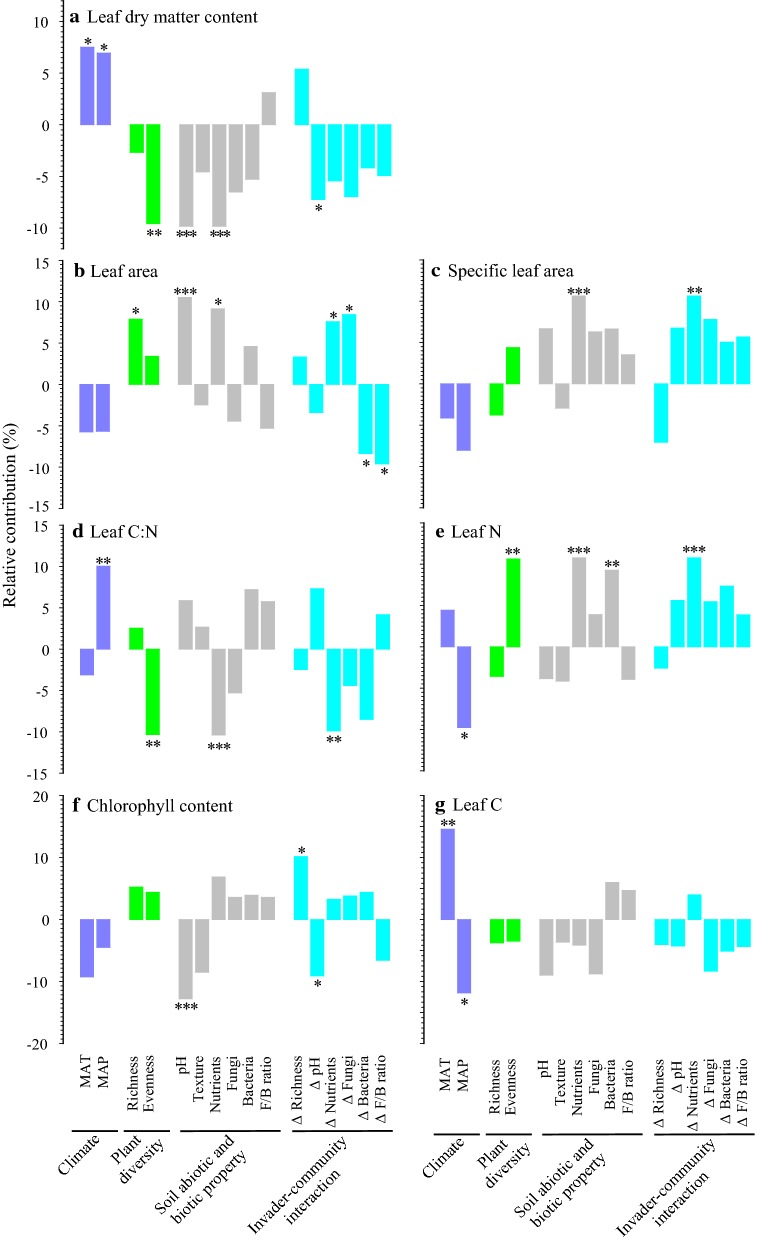
The relative contributions of 16 environmental factors to the variation in leaf dry matter content (**a**), leaf area (**b**), specific leaf area (**c**), leaf C:N ratio (**d**), leaf N (**e**), leaf chlorophyll index (**f**), and leaf C (**g**). The positive/negative direction of each effect is indicated by its positive/negative value. ***P < 0.001; **P < 0.01; *P < 0.05

Note that the per capita contribution of climate, native plant diversity, soil properties or *S. canadensis*–community interactions (i.e., the mean contribution of all explanatory variables) was used for comparing their relative contributions. The per capita contribution of climate, diversity, soil, and interactions varied with LDMC (Fig. 2a), leaf area (Fig. 2b), SLA (Fig. 2c), leaf C:N (Fig. 2d), leaf N (Fig. 2e), chlorophyll (Fig. 2f), and leaf C (Fig. 2g); the dominant factors underlying trait variation also varied with leaf traits (Fig. 2a–g). Across the seven leaf traits, the per capita contribution ranked: climate (7.5%) > soil (6.2%) > interactions (6.1%) > diversity (5.4%). For climate, MAP (8.1%) contributed to leaf trait variation more than MAT (7.0%); for native plant diversity, native plant evenness (6.6%) contributed to leaf trait variation more than plant richness (4.2%); for soil properties, soil nutrient availability (8.8%) was the most important contributor to leaf trait variation; for *S. canadensis*–community interactions, the change in soil nutrients (7.4%) was the most important contributor to leaf trait variation (Fig. 2a–g). Across the seven leaf traits, the total contribution of biotic variables to leaf trait variation (49.0%) was close to that of abiotic variables (51.0%).

LDMC increased with climate but decreased with diversity, soil, and interactions (Fig. 2a). Leaf area increased with diversity and soil, and it increased or decreased with interactions (Fig. 2b). SLA increased with soil and interactions (Fig. 2c). Leaf C:N ratio increased with climate but decreased with diversity, soil, and interactions (Fig. 2d); however, the opposite was the case for leaf N (Fig. 2e). Chlorophyll decreased with soil pH, and it increased or decreased with interactions (Fig. 2f). Leaf C was influenced by MAT and MAP, both of which had opposing effects on leaf C (Fig. 2g).

**Fig. 3:**
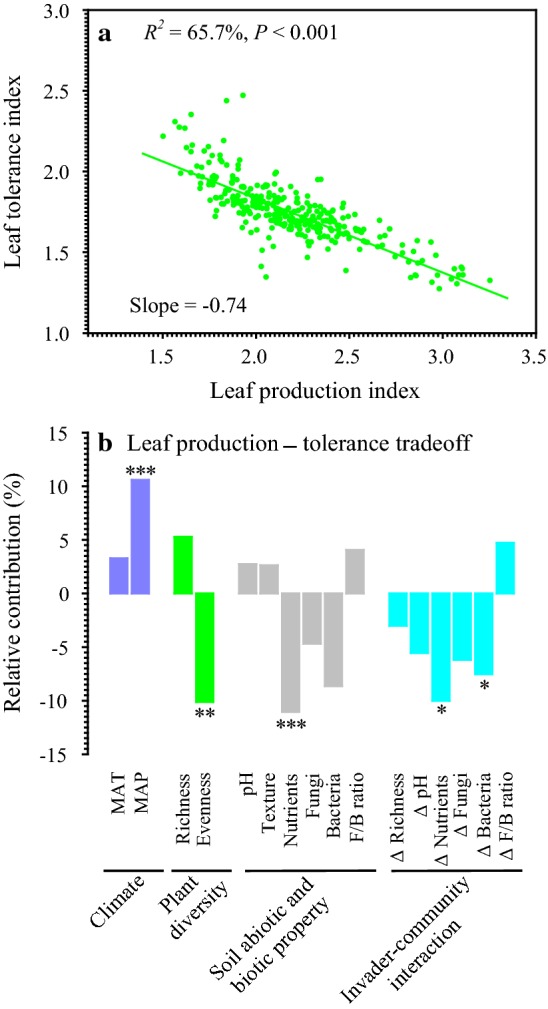
The relationship between leaf production index and leaf stress-tolerance index (**a**) and the relative contributions of 16 environmental factors to leaf production-tolerance trade-off (**b**). The positive/negative direction of each effect is indicated by its positive/negative value. ***P < 0.001; **P < 0.01; *P < 0.05

The seven leaf traits were highly interrelated (Additional file 1: Fig. S1), and these leaf traits were functionally categorized into two categories: leaf production potential (incorporating leaf area, SLA, chlorophyll, and leaf N) and leaf stress-tolerance potential (incorporating LDMC, leaf C, and leaf C/N). There was a significantly negative correlation between leaf production potential and leaf stress-tolerance potential, and 65.7% of the total variation was explained by each other (Fig. 3a). For this trade-off between leaf production and leaf stress-tolerance, the per capita contribution followed the order: diversity (7.7%) > climate (6.9%) > interactions (6.2%) > soil (5.6%); the most key contributor was soil nutrients (11.0%) among 16 variables (Fig. 3b). Interestingly, climate contributed positively to this trade-off whereas diversity, soil, and interactions contributed negatively to it. In other words, climate played a positive role in shaping the relationship between leaf production and leaf tolerance, and diversity, soil, and interactions followed the opposite direction.

## Discussion

### Leaf trait variation and its drivers

We found that the leaves of invasive *S. canadensis* exhibited high phenotypic variation and there were substantial differences in variation among the seven leaf traits (PVI ranging from 0.696 to 0.427). Surprisingly, leaf physiological traits such as leaf chlorophyll and N had lower variation relative to leaf size and thickness. In other words, fast response variables were less sensitive to ecological gradients than slow response variables. The contribution of climate to leaf variation was greatest while the contribution of native plant diversity was smallest. This might be among the reasons why plant ecologists have emphasized broad relationships between leaf traits and climate for at least a century [9]. Overall, MAP contributed to leaf variation more than MAT, and soil nutrients and their changes contributed greatly to leaf variation. Thus, water and nutrient resources played a key role in shaping leaf variation. In addition, the dominant factor(s) varied with leaf trait identities. For example, species evenness dominated over richness for LDMC, leaf N, and leaf C:N, and the opposite was the case for leaf area. Such phenomena also occurred in soil properties and *S. canadensis*–community interactions.

Altitude affects plant trait variation [19, 27], but we did not consider this factor due to the small change in elevation. Nor did we consider light because all the leaves measured in our study were exposed to full sunlight. Our results do not support the leading paradigm that trait variation across geographic areas seems more related to abiotic factors [9, 17–19] because abiotic and biotic factors contributed similarly to trait variation based on their relative contributions. Thus, the role of biotic environments cannot be overlooked when addressing the mechanisms underlying trait variation. One important corollary of our results is that exploring the way plant traits vary across environmental gradients might help us to understand the functioning of trait variation in a changing world.

We found that climate, diversity, soil, and interactions had contrasting directions in regulating leaf traits. More specifically, their directions were commonly different but not the same consistently. Additionally, the directions of climate, diversity, soil, and interactions varied with focal leaf traits. For example, climate exhibited positive, neutral or negative effects depending on the identity of leaf traits. Such phenomena also occurred in diversity, soil, and interactions. For a given category of environmental factors, there were some differences in direction among different variables. For instance, MAT and MAP differentially influenced leaf C, N, and C:N ratios. Taken together, these patterns are intriguing, and the related mechanisms deserve more attention.

### Leaf trait spectrum and its drivers

As opposed to our hypothesis that the leaf production potential of *S. canadensis* is positively associated with its leaf stress-tolerance potential along ecological gradients, there was a negative correlation (also called as a trade-off) between them. Interestingly, our recent study found that leaf production potential was positively associated with leaf stress-tolerance potential across 107 woody species grown in common botanical gardens [26]. This trade-off was primarily controlled by soil properties and *S. canadensis*–community interactions, and the contributions of climate and native plant diversity to this trade-off were relatively low. These findings provide a potential explanation why the contribution of climate to the worldwide leaf economics spectrum is quite modest [9]. Leaf trait spectra have been commonly ascribed to abiotic factors such as climate and soil fertility [28] so that the role of biotic factors has been neglected. Our findings suggest native plant diversity and invader–community interactions (e.g., changes in soil bacteria and fungi) play a key role in shaping leaf trait spectra. These results appear to be reasonable, because biotic environments are among the core components of selective pressures [2, 10].

We observed that climate contributed positively to this leaf trait spectrum while diversity, soil, and interactions contributed negatively to it. Specifically, climate exhibited facilitative effects on the leaf production-tolerance trade-off, and diversity, soil, and interactions showed inhibitive effects on this trade-off. However, it should be noted that the facilitation of climate was mainly ascribed to precipitation rather than air temperatures. In addition, evenness and soil nutrient availability played an overwhelming role in inhibitive effects.

The trade-off between leaf production potential and stress-tolerance potential delivers several implications. For example, plant species occupying resource-rich habitats are characterized by higher resource uptake and biomass production but lower stress-tolerance than their resource-poor counterparts [8–11]. Identifying holistic responses and disentangling the relative importance of suites of causal factors are crucial for understanding pairwise relations and systematic trends of plant traits along multiple biotic and abiotic gradients. Additionally, root traits should be paid more attention when addressing root–leaf trait spectra because of their importance in responding to belowground stimuli, although the root trait variation and spectra were not shown explicitly in this study due to relatively limited root samples.

## Conclusions

In our study, 16 biotic and abiotic factors shaping leaf trait variation and spectra were considered at the same time, although we cannot provide a complete picture covering the gamut of biotic and abiotic drivers. This study provides a solid basis for understanding how climate, native plant diversity, soil properties, and interactions between invasive species and recipient communities influence trait variation and spectra among populations, and also highlights the relative importance of multiple determinants in shaping trait variation and spectra. Our results have several implications. First of all, multiple plant traits and trait spectra should be considered simultaneously because they have different responses to the same drivers. Second, biotic factors and interactions between target plants and their surrounding communities might play a key role in shaping trait variation and spectra. This aspect remains poorly understood. Finally, leaves have high resource acquisition and production but low stress-tolerance at one end of the economics spectrum, and the opposite is the case at the other end [8, 9]. This spectrum reflects whole-plant strategy shifts in leaf traits along multiple gradients and helps us to better understand invasion success.

## Methods

### Study species and region

*Solidago canadensis* L. (Compositae) is a perennial forb native to North America; it can produce seeds and rhizomes simultaneously, and can dominate or even form monocultures in some habitats [29]. In its home range, *S. canadensis* has a broad climatic tolerance and is commonly found in moist situations or on soils classed as “medium” in both texture and organic matter content, although it is also found on muck soils [30].

**Fig. 4:**
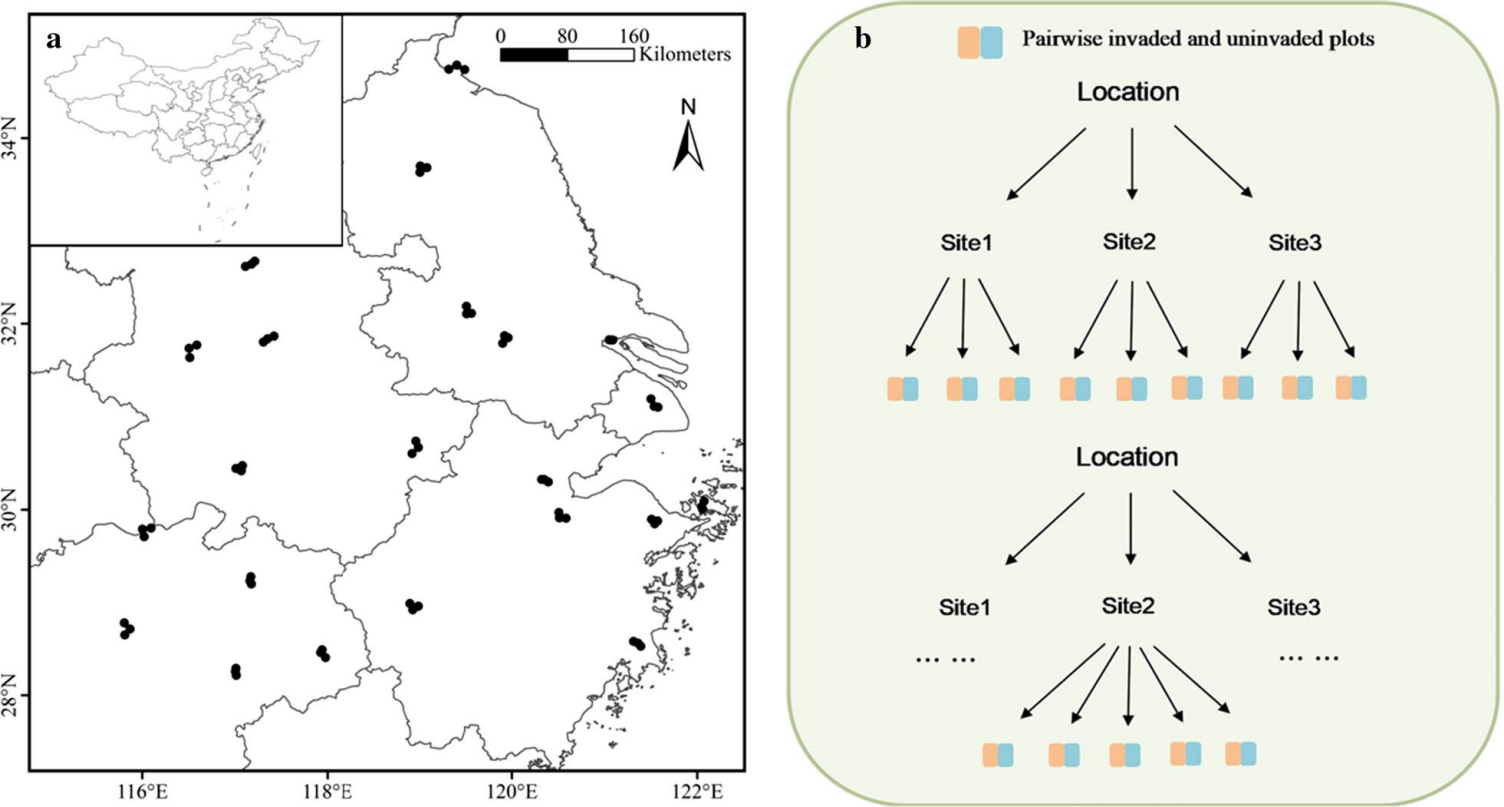
An illustration of 22 sampling locations and 66 sampling sites in our study (**a**) and plot layout (**b**)

*Solidago canadensis* was introduced into China as an ornamental plant in the 1935 [31] and now it has invaded large areas of southern China [25]. This invaded range belongs to a subtropical climate. Our study region roughly covered an area of 800 × 800 km (Fig. 4a), and the altitudinal difference was 76 m across the entire study region (ranging from 3 to 79 m). The related physical information is presented in Additional file 1: Table S1. To date, little is known about what drives its leaf trait variation and spectra across an entire invaded range in China.

In the invaded range, we selected 22 sampling locations, each with three sites (Fig. 4a, b). Specifically, we surveyed five pairs of 1 × 1 m plots (i.e., invaded and uninvaded plots) at 51 sites and three pairs of 1 × 1 m plots at 15 sites based on the field conditions (Fig. 4b). Invaded plots had high *S. canadensis* cover. Paired plots were identified according to the following two criteria [32]: they were proximate (i.e., 2–5 m interval) and occurred under similar soil and topographic conditions; they had the same subdominant native species. Thus, 300 pairs of invaded and uninvaded plots were chosen. In other words, we sampled 66 different *S. canadensis* populations across its invaded range in China.

### Determinations of leaf traits

To determine leaf traits, we randomly collected 30 leaves from five different individuals per population, and measured their leaf area, specific leaf area (SLA), leaf dry matter content (LDMC), leaf chlorophyll, leaf C concentration, leaf N concentration, and leaf C:N ratio. All leaves were collected from the top canopy of *S. canadensis* communities (i.e., exposed to full sunlight).

Leaf area was determined in situ with an area meter, water-saturated fresh leaf mass was determined after rehydrating leaves at room temperature for 12 h, and dry leaf mass was determined after oven-drying at 85 °C for 48 h. We calculated SLA and LDMC as follows:

1 $$ {\text{SLA}} = \frac{Leaf\; area}{Dry\; biomass} \quad \left( {{\text{cm}}^{2} {\text{g}}^{ - 1} } \right) $$

2 $$ {\text{LDMC}} = \frac{Dry \;biomass}{Fresh \;biomass} \quad \left( {{\text{mg g}}^{ - 1} } \right) $$

We measured leaf chlorophyll index in situ using a SPAD meter. The oven-dried leaves were ground for measurements of leaf C and N using an elemental analyzer (vario EL III). We calculated the ratio of leaf C to N.

### Determinations of 16 biotic and abiotic factors

To quantify the relative contributions of biotic and abiotic variables to trait variation and spectra, we selected four sets of factors: climate, native plant diversity, soil properties, and *S. canadensis*–community interactions. Climate included the mean annual temperature (MAT) and mean annual precipitation (MAP), which were collected from the nearest weather stations of sampling sites. We selected 300 pairwise uninvaded and invaded plots and determined native plant diversity and soil properties therein. Note that the 66 *S. canadensis* populations measured for leaf traits were from the invaded plots. Native plant diversity included native species richness and Pielou evenness index, which were calculated as described by Dong et al. [25]. To calculate species evenness, we first quantified the relative abundance of species, which was determined based on the numbers of individuals per species in a plot. Soil properties included pH, texture, nutrients, bacteria, fungi, and fungi/bacteria ratio. We measured soil pH in a soil solution rate of 1:2.5 (soil:distilled water) using a pH meter (Sartorius PB-10 m), soil texture (i.e., clay%:silt%:sand%) using a laser particle size analyzer (Mastersizer 2000), soil available phosphorus (AP) using a UV-2550 ultraviolet spectrophotometer, and soil ammonia (NH_4_-N) and nitrate (NO_3_-N) using a continuous flow analyzer. Soil nutrients referred to the sum of AP, NH_4_-N, and NO_3_-N because they exhibited strong collinearity. We determined soil bacteria and fungi using the phospholipid fatty acid analysis (see Dong et al. [24, 25] for details) and calculated the ratio of fungi to bacteria.

To quantify the *S. canadensis*–community interactions, we calculated the relative change (Δ) in native plant diversity (i.e., Δrichness), soil abiotic properties (i.e., ΔpH and Δnutrients), and soil microbes (i.e., Δfungi, Δbacteria, and ΔF/B ratio). The relative change was calculated as follows:

3 $$ \Delta = \frac{Vi - Vu}{Vi + Vu} $$

where *Vi* and *Vu* represent a given functional trait in the pairwise invaded and uninvaded plots, respectively.

### Data analyses

Thirty leaves per plot were sampled so that their values were averaged as a proxy of each plot. To quantify the variation in each *S. canadensis* trait, we proposed a phenotypic variation index (*PVI*):

4 $$ PVI = \frac{{Max \left( {Ti} \right) - Ti}}{{Max \left( {Ti} \right) - Min \left( {Ti} \right)}} $$

where *Ti* represents a given trait of the *i*th population (i = 1, 2, …., 300), and *Max (Ti)* and *Min (Ti)* represent the maximum and minimum values of a specific trait among 66 populations. We categorized seven leaf traits into two categories: leaf production traits and leaf stress-tolerant traits [26]. Accordingly, we proposed a leaf production index (LPI) and leaf stress-tolerance index (LTI) as follows:

5 $$ {\text{LPI}} = \mathop \sum \limits_{j = 1}^{4} \frac{Ti}{{Max \left( {Ti} \right)}} $$

6 $$ {\text{LTI}} = \mathop \sum \limits_{j = 1}^{3} \frac{Ti}{{Max \left( {Ti} \right)}} $$

where *Ti* represents a given trait of the *i*th population (i = 1, 2, …., 300); for LPI, *j* represents four leaf production traits: leaf area, SLA, chlorophyll, and leaf N; for LTI, *j* represents three leaf tolerance traits: LDMC, leaf C, and leaf C/N ratio. The raw data on leaf traits are presented in Additional file 2.

We used a one-way analysis of variance with Post Hoc *Tests* to test whether there were differences in phenotypic variation among seven leaf traits. Note that this test was not used for the relative contributions of different determinants to trait variation and trait spectra (see below). Bivariate relationships of log-transformed leaf traits were first assessed with model II linear regression and with standardized major axis line fits [33]. Specifically, a model II regression was used to test the relationships among seven leaf traits and between LPI and LTI. Note that the ratio of LTI to LPI was calculated to quantify each trait spectrum.

To assess the relative contributions of environmental variables to trait variation and spectrum, we selected the multimodel inference approach, which is based on all the models in a priori set, not just the one estimated to be best, and therefore can provide more stable and reliable inference results than traditional statistical inference [34].

The global models included one dependent variable (i.e., variation in each trait or LTI/LPI) and 16 explanatory variables (i.e., MAT, MAP, richness, evenness, pH, texture [i.e., clay/silt/sand ratio], nutrients, bacteria, fungi, F/B ratio, Δrichness, ΔpH, Δnutrients, Δbacteria, Δfungi, and ΔF/B ratio). It should be noted that plant diversity and soil properties referred to those in uninvaded plots rather than in invaded plots because they roughly represent the initial regimes of recipient communities. We used the model selection method to generate all possible candidate models from the global models, and then all the candidate models were ranked according to the second-order Akaike’s information criterion. The effect size of each variable was expressed by the averaged model parameters deriving from accumulated model probability exceeded 95%. The importance of each variable was estimated by summing the Akaike’s weights of each model [34, 35]. The relative contribution of a given variable was estimated through dividing its importance by the total importance of 16 variables. The total contribution of climate, native plant diversity, soil properties or *S. canadensis*–community interactions equaled the sum of the contributions of the corresponding variables, and the per capita contribution of climate, native plant diversity, soil properties or *S. canadensis*–community interactions was estimated through dividing the total contribution by the numbers of corresponding variables.

All statistical analyses were performed using R version 3.5.2 [36]. The MMI approach was performed using *dredge* function in the package “MuMIn” [37].

## Additional files

**Additional file 1: Figure S1.** Bivariate relationships between seven leaf traits. Lines represent Model II regressions. **Table S1.** Geographic information about sampling locations.

**Additional file 2** The raw data on leaf traits.

## Data Availability

All data generated or analysed during this study are included in this published article and its supplementary information files or are available from the corresponding author on reasonable request.

## Abbreviations

SLA: specific leaf area
LDMC: leaf dry matter content
C: carbon concentration
N: nitrogen concentration
MAT: mean annual temperature
MAP: mean annual precipitation
AP: the concentration of soil available phosphorus
NH_4_-N: the concentration of soil ammonia
NO_3_-N: the concentration of nitrate
PVI: phenotypic variation index
LPI: leaf production index
LTI: leaf tolerance index
