# Supplementary material for: The relative contributions of climate, soil, diversity and interactions to leaf trait variation and spectrum of invasive Solidago canadensis
Source: BMC Ecol. 2019 Jun 15;19:24. doi: 10.1186/s12898-019-0240-1 (PMC6570854; doi:10.1186/s12898-019-0240-1)
Supplement: Supplementary file 1 — Additional file 1: Figure S1. Bivariate relationships between seven leaf traits. Lines represent Model II regressions. Table S1. Geographic information about sampling locations. [file 12898_2019_240_MOESM1_ESM.docx]

**Supplementary materials**

**Figure S1** Bivariate relationships between seven leaf traits. Lines represent Model II regressions.

**Figure S1**

**Table S1** Geographic information about sampling locations

| Location | Altitude (m) | Latitude | Longitude | Temperature  (°C) | Precipitation  (mm) |
| --- | --- | --- | --- | --- | --- |
| Lianyungang | 31 | 34°44'N | 119°20'E | 14.9 | 1025 |
| Huaian | 40 | 33°37'N | 119°04'E | 15.1 | 1049 |
| Zhenjiang | 15 | 32°08'N | 119°33'E | 16.2 | 1101 |
| Changzhou | 16 | 31°49'N | 119°56'E | 16.5 | 1077 |
| Nantong | 21 | 31°49'N | 121°05'E | 16.2 | 1089 |
| Shanghai | 3 | 31°06'N | 121°34'E | 17.4 | 1112 |
| Hangzhou | 10 | 30°19'N | 120°23'E | 17.6 | 1324 |
| Zhoushan | 4 | 30°00'N | 122°03'E | 17.1 | 1286 |
| Shaoxing | 44 | 29°54'N | 120°30'E | 17.6 | 1374 |
| Ningbo | 12 | 29°50'N | 121°32'E | 17.8 | 1394 |
| Quzhou | 70 | 28°55'N | 118°55'E | 17.9 | 1503 |
| Taizhou | 73 | 28°33'N | 121°21'E | 18.4 | 1654 |
| Shangrao | 74 | 28°27'N | 117°55'E | 18.2 | 1779 |
| Yingtan | 53 | 28°12'N | 117°00'E | 18.9 | 1878 |
| Jingdezhen | 39 | 29°16'N | 117°10'E | 18.4 | 1695 |
| Nanchang | 44 | 28°42'N | 115°52'E | 18.6 | 1520 |
| Jiujiang | 38 | 29°44'N | 116°01'E | 18.1 | 1310 |
| Anqing | 19 | 30°28'N | 117°04'E | 17.5 | 1322 |
| Ningguo | 54 | 30°40'N | 118°59'E | 16.4 | 1292 |
| Hefei | 35 | 31°48'N | 117°18'E | 16.6 | 1031 |
| Luan | 73 | 31°44'N | 116°30'E | 16.4 | 1133 |
| Huainan | 43 | 32°37'N | 117°06'E | 16.8 | 982 |
